# Supplementary material for: A Global Screen for Assembly State Changes of the Mitotic Proteome by SEC-SWATH-MS
Source: Cell Syst. 2020 Feb 26;10(2):133–155.e6. doi: 10.1016/j.cels.2020.01.001 (PMC7042714; doi:10.1016/j.cels.2020.01.001)

Q86YS7 | C2CD5_HUMAN | C2CD5 CDP138 KIAA0528
Monomer MW [kDa]: 110.447 Monomer expected elution fraction: 39

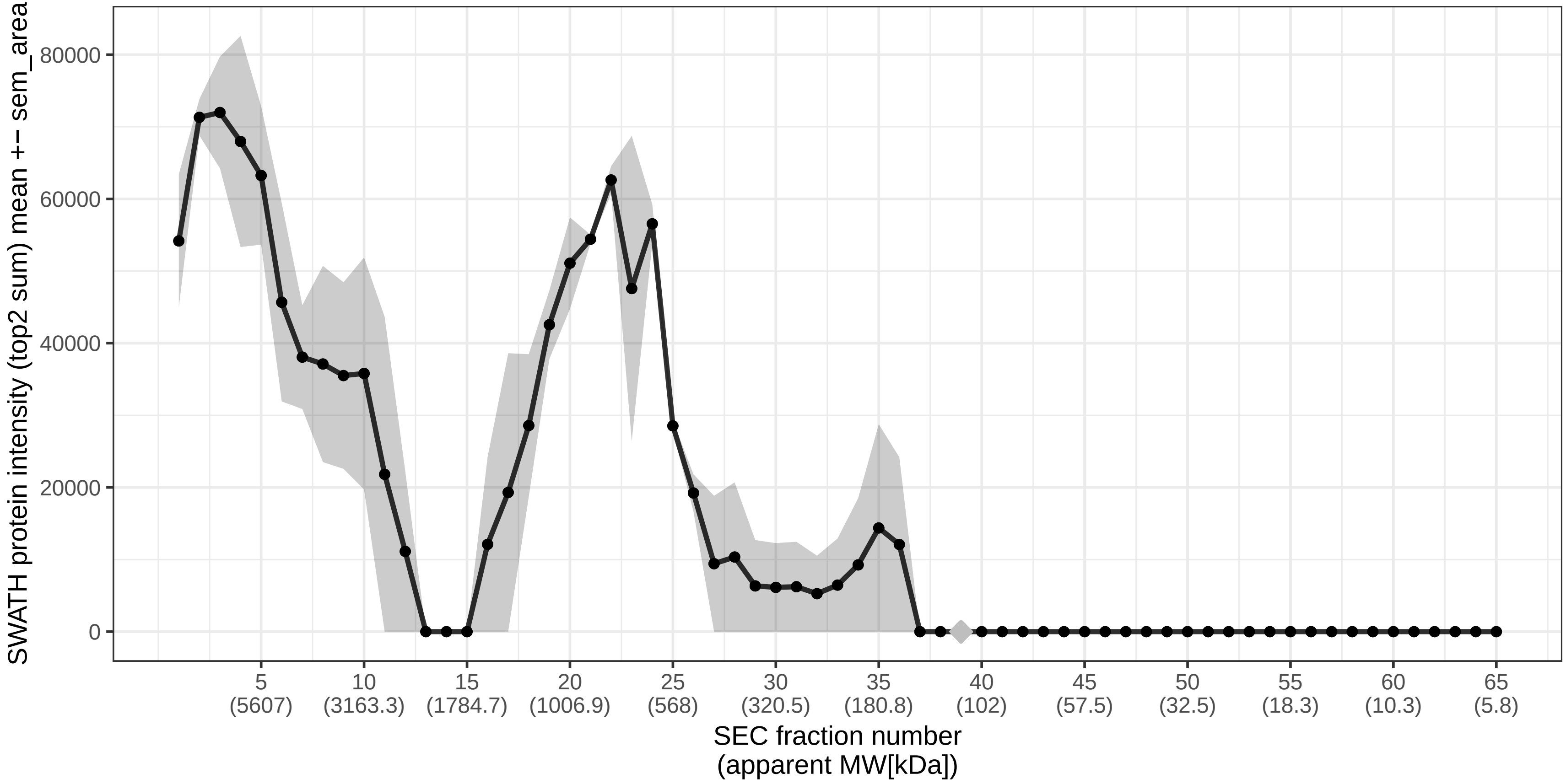

Supplement: Data S1. SEC-SWATH-MS Protein Chromatograms, Related to Figure 1 [file mmc6.zip › SECchrom_Q86YS7_C2CD5_HUMAN_C2CD5_CDP138_KIAA0528.pdf]
